# Trust me, you are there: the role of verbal manipulation on embodiment and body localization

**DOI:** 10.1007/s00426-026-02271-z

**Published:** 2026-04-11

**Authors:** Francesca Frisco, Vito Bruno, Daniele Romano, Giorgia Tosi

**Affiliations:** 1 Department of Psychology, University of Milan-Bicocca, Piazza dell’Ateneo Nuovo 1, Milan, 20126 Italy; 2 Milan Center for Neuroscience, Milan, Italy; 3 Mind and Behavior Technological Center, University of Milan-Bicocca, Milan, Italy

**Keywords:** Virtual RHI, Body ownership, Body’s location, Verbal manipulation

## Abstract

Body ownership (the perception of the body as belonging to oneself) and body spatial perception arise from multisensory integration and can be altered by body illusions (e.g., Rubber Hand Illusion, RHI). In the RHI, synchronous visuo-tactile stimulation elicits ownership over a fake hand and shifts the perceived real hand position (Proprioceptive Drift). This study investigated whether altering body spatial predictions through verbal manipulation affects ownership in a virtual RHI (vRHI). In the Misleading Information Condition, participants were told their hand would be moved closer to the virtual hand. In the Correct Information Condition (Control Condition), participants were told their hand would be moved and then put back in its original position. Embodiment and perceived hand position were assessed at baseline (T0), after verbal manipulation (T1), and after vRHI induction (T2). Results showed that verbal manipulation altered spatial predictions: in the Misleading Information Condition, proprioceptive drift at T1 increased. However, after the vRHI, the drift was lower in the Misleading Information Condition than in the Control condition. Embodiment increased only after the vRHI. Thus, manipulating spatial predictions influenced hand position but not embodiment, revealing top-down modulation of body spatial perception. In contrast, multisensory illusion affected perceived position and embodiment, independently of prior verbal manipulations, suggesting a possible distinction between these processes.

**Supplementary Information:**

The online version contains supplementary material available at 10.1007/s00426-026-02271-z.

## Introduction

The sense of body ownership (i.e., the experience of the body as belonging to oneself) and the perception of one’s body location in space mainly rely on the multisensory and sensorimotor integration of incoming stimuli (Blanke, 2012; Ehrsson, 2019; Longo et al., 2010). These two aspects are pivotal components of bodily self-consciousness (Serino et al., 2013), essential for effective interactions with the external world. However, how the sense of body ownership and the perceived spatial location of the body are associated is still a matter of debate in the literature. For instance, this relation has been studied in the established Rubber Hand Illusion (RHI; Botvinick & Cohen, 1998), where a rubber hand is placed near the hidden real hand, and a paintbrush simultaneously strokes both. In this illusion, participants often feel ownership over the rubber hand, reduced ownership over their real hand (i.e., disownership), and a perception that their hidden hand is now closer to the rubber hand (i.e., proprioceptive drift). The anatomical plausibility of the fake hand position and the visuo-tactile congruency during the RHI induce ownership feelings over the rubber hand. Simultaneously, the discrepancy between the real and fake hand position causes the recalibration of the body’s spatial position and the emergence of disownership feelings (Longo et al., 2008; Romano et al., 2021).

While some studies suggest distinct mechanisms underlying proprioceptive drift and ownership sensations, as measured by questionnaires, others argue for a relationship between them (Makin et al.,^2008^; Rohde et al., 2011; Kalckert & Ehrsson, 2012; Lira et al., 2017). A recent meta-analysis has shed light on this debate (Tosi et al., 2023), revealing the effective presence of a correlation between the two indices. This finding indicates that proprioceptive drift and ownership sensations are associated, even if measuring different aspects of the RHI phenomenon.

We have recently proposed that the sense of body ownership would arise from where our body is perceived (Tosi & Romano, 2023). We hypothesized that if the body is predicted to be at a specific location, a fake body placed at that spot is likely to be felt as one’s own. This hypothesis is based on previous evidence that showed that a mismatch between predicted and actual locations can elicit disownership of the real body. For instance, in the RHI, the predicted location conflicts with the real hand’s position, producing ownership over the fake hand and proprioceptive drift but also disownership of the real hand (Longo et al., 2008; Romano et al., 2021). On the contrary, when the real and the fake body are aligned, ownership seems to emerge without disownership (Tosi et al., 2022). Taken together, these observations support a location-to-ownership direction hypothesis, whereby variations in perceived location precede and modulate ownership. In a previous study, we specifically investigated the link between ownership feelings and the perception of the body’s location in space (Frisco et al., 2024). Participants were exposed to the virtual version of the RHI (vRHI) or a first-person perspective Full Body Illusion-like paradigm (1pp-FBI). In both illusions, the virtual body could be aligned or misaligned with the real body. Considering the 1pp-FBI, we found evidence supporting that the perceived body location could be associated with the emergence of ownership feelings. Indeed, while ownership feelings for the virtual avatar always emerged after the illusion, the perceived body location shifted towards the avatar only when the virtual body was not aligned with the real body, along with an increase in disownership sensations. Also, we found a correlation between the perceived leg’s position and ownership feelings when a position recalibration was needed (i.e., when the virtual body was not aligned with the real body).

Accordingly, in the present study, we aimed to investigate further the role of body spatial predictions in shaping the emergence of body ownership and disownership sensations. We predict that body ownership and body spatial predictions are so closely related that alterations in the perceived body position may influence body ownership feelings. In particular, by employing verbal manipulation (i.e., brief instructional statements regarding the change in body position), we specifically changed the prediction of body location in space to alter the sense of body ownership. Thus, embodiment illusions may arise not only from multisensory integration but also from participants’ body spatial predictions implicitly shaped by verbal information. Accordingly, we used verbal information to manipulate spatial predictions and influence prior beliefs about body location, thereby engaging top-down mechanisms that may modulate ownership sensations. Changes in body ownership and body spatial predictions were assessed after a visual exposure to the virtual body (T0), after the verbal manipulation and a second visual exposure (T1), and after the vRHI induction (T2).

First, we expected a change in the predictions of hand location in T1 only after the Misleading Information Condition and not after the control condition, as only the first condition was designed to change belief about the spatial position. This manipulation aimed to bias participants’ spatial expectations without altering the actual hand position. Moreover, we expected that the perceived increased proximity between the real and virtual hands, induced during the Misleading Information Condition, should enhance ownership feelings over the virtual hand and disownership sensations over the real hand, as spatial proximity has been consistently associated with stronger embodiment responses in prior literature (Lloyd, 2007). Second, after the vRHI induction (i.e., T2), we expected stronger ownership and disownership sensations with a further displacement of the perceived hand position towards the virtual hand compared to T1. This hypothesis is supported by previous studies showing that synchronous visuotactile stimulation induces body ownership sensations and shifts in perceived hand position (e.g., Longo et al., 2008; Romano et al., 2021), which, in this case, may be further reinforced by the spatial biases introduced through the misleading verbal manipulation. In addition, considering that Misleading Information about the body position can increase the probability that participants perceive their hand closer to the avatar, we expected the perceived position in T1 to predict ownership feelings in T2. This would directly test our core hypothesis that ownership sensations can be influenced by modulations in the spatial predictions about the body.

## Materials and methods

This work, including hypotheses, design, sampling, and analysis plan, was preregistered at the following link: https://osf.io/xqjpg/?view_only=c3eae36accc1403093793ca12fd2b85e.

### Participants

Eighty-one healthy participants were recruited in the study (48 female, mean age: 22.66 ± 3.22 years; mean school age: 14.24 ± 1.90 years). Participants’ recruitment took place between December 2023 and March 2024. All participants either had normal vision or vision corrected to normal and were not aware of the experiment’s objectives. Before taking part, participants provided written informed consent. The local Ethics Committee at the University of Milano-Bicocca approved the study (RM-2022-574), which follows the ethical guidelines of the Declaration of Helsinki (World Medical Organization, 1996). The experimenter explained the study’s general objectives and procedures to participants before giving consent. At the end of the experiment, participants were informed in detail about the study’s aims. The sample size was determined to find a significant correlation between ownership and proprioceptive drift, according to the prior meta-analysis results on the RHI (Tosi et al., 2023), and to detect a mean difference in a bodily illusion paradigm with a within-subject experimental design. The previous meta-analysis (Tosi et al., 2023) revealed that a sample size between 46 and 69 participants is necessary to investigate correlation effects effectively. In this work, the authors reported robust evidence supporting a correlation between subjective embodiment and proprioceptive drift (*r* = 0.35, 95% CI = [0.285, 0.415], r² = 0.12; BF = 1.07e + 9). Based on this estimate, a power analysis using α = 0.05 and power = 0.80 indicated that a minimum of 46 participants would be required to detect a significant effect. However, to account for the possibility that publication bias may have inflated the observed effect size (Perugini et al., 2014), a more conservative power analysis was also conducted using the lower bound of the confidence interval (*r* = 0.29). This analysis yielded a recommended sample size of 69 participants. A priori sample size was also computed for a within-subject ANOVA design using G*power to test the embodiment effect in a bodily illusion paradigm. Considering a power of 0.80, an alpha of 0.05, and an effect size of f = 0.65 (η^2^_p_ = 0.30; Tosi et al., 2023, 2022), the analysis suggested a necessary sample of 22 participants. According to the power analysis and the potential overestimation of findings from meta-analytic studies, we planned to recruit a sample size of 80 participants. This target exceeds the most conservative requirement based on correlation analysis (*n* = 69), ensuring adequate power in the presence of potential exclusions (e.g., technical failures).

### Procedure and experimental design

#### Procedure and apparatus

**Fig. 1:**
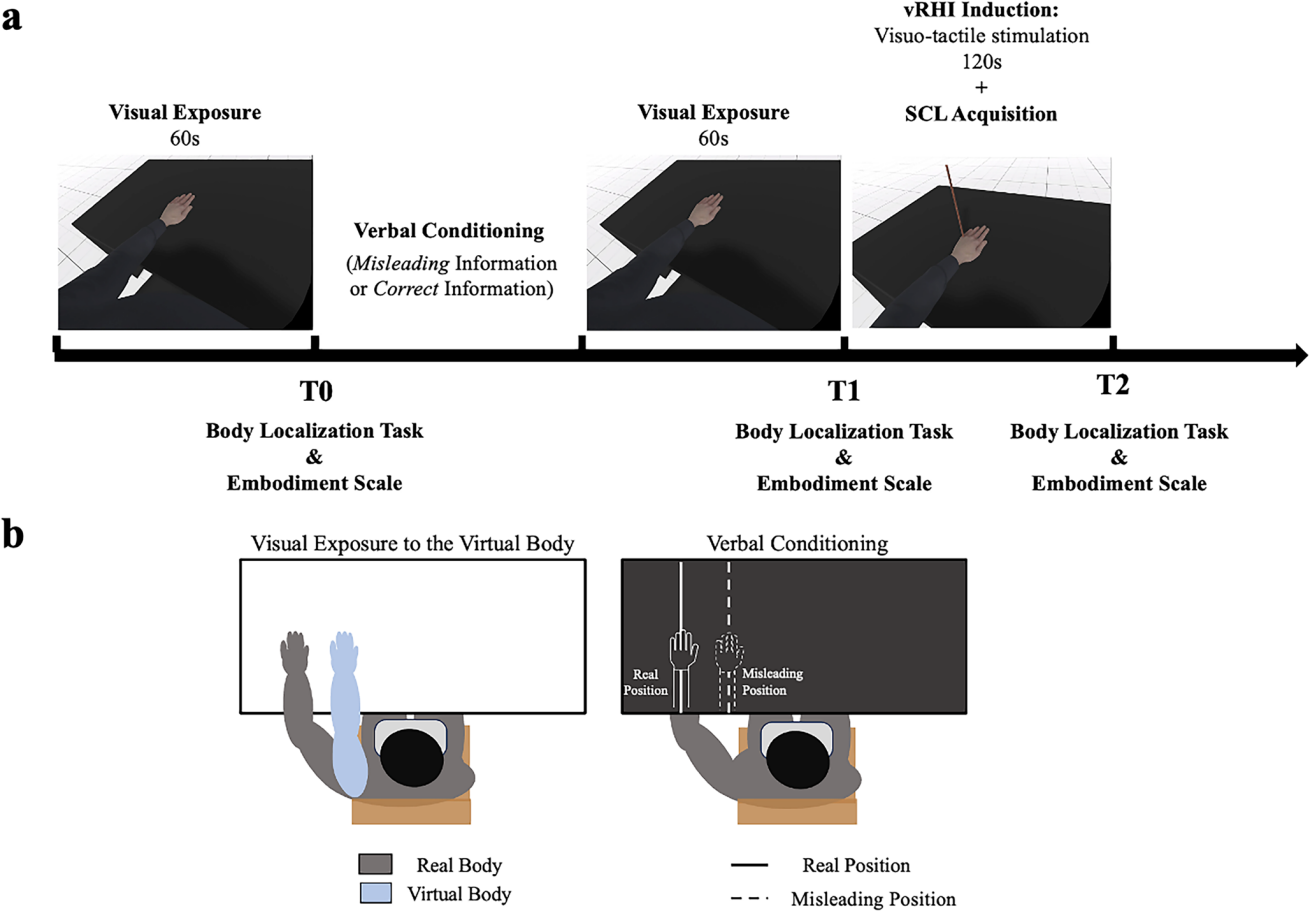
Experimental design and procedure. **(a)** The upper panel shows the procedure timeline. First, participants observed the virtual body for 60 s (i.e., visual exposure). After the verbal manipulation (counterbalanced across participants), participants observed again the virtual body for 60 s. Finally, participants were exposed to the vRHI: after 120 s of visuo-tactile stimulation, a virtual knife stabbed the virtual hand. The skin conductance level (SCL) was collected. After each phase, participants first performed a Body Localization Task and then completed the Embodiment Scale (T0, T1, and T2). **(b) **The lower panel shows the hands’ placement during the visual exposure and the vRHI: the real hand is depicted in grey, and the virtual hand is depicted in blue; the right panel shows the hands’ placement during the verbal manipulation: participants always kept their hand in the position of the solid line. In the Misleading Information Condition, participants were told that their left hand would be moved towards the dashed line; however, the hand was temporarily displaced and then positioned in the original location (i.e., on the solid line). In the Correct Information Condition, participants were told their left hand would be temporarily displaced and relocated to the original position (i.e., on the solid lines) vRHI= virtual Rubber Hand Illusion; SCL= Skin Conductance Level

Participants were exposed to a vRHI, and verbal manipulation aimed to alter the spatial predictions of their real hand (Fig. 1a). The experimental procedure consisted of three distinct time points (T0, T1, and T2) across two verbal instruction conditions (Misleading Information Condition vs. Correct Information Condition). Participants sat on a chair with their left hand placed palm-down on the table. The hand was placed 24.0 cm from the torso’s midline towards the left. The right hand was positioned on the participant’s leg under the table and equipped with electrodes to measure skin conductance level (SCL). Participants wore the Head Mounted Display (HMD; Oculus Quest 2), through which the virtual environment was presented using Unity 2022 Software by connecting the HMD to the computer (OMEN 30 L Desktop GT13-0xxx, Intel Core i9-10900 K, 64GB RAM, NVIDIA GeForce RTX 3090). The virtual environment consisted of a neutral space with an avatar sitting on a chair at a table, mimicking the participants’ position. The avatar’s head was substituted with the camera so that the participant’s point of view corresponded to the avatar’s eyes. The virtual hand was shifted 18.6 Unity centimetres to the right of the real hand’s position. The shift in the virtual hand’s position corresponded to an estimated change of 14.0 cm in the real environment (see Fig. 1b, left panel).

Participants first observed the virtual body for 60 s. Then, the perceived hand location was assessed (T0) through a Body Localization Task, and subsequently, embodiment sensations (i.e., ownership and disownership) were measured using the Embodiment Scale (Romano et al., 2021). Subsequently, the virtual body was always covered by a black virtual plane (see Fig. 1b, right panel), and we used verbal manipulation in two possible conditions, with the order counterbalanced across participants. In the experimental condition (i.e., Misleading Information Condition), participants were told that their actual hand would be moved to the right, closer to the body midline (i.e., closer to the virtual hand’s location; dashed line in the right panel of Fig. 1b). The experimenter then asked participants to temporarily move their left hand and keep it relaxed on their legs. After 30 s, the experimenter put the participant’s left hand back in the original position (i.e., the same position as in T0, as shown by the solid line in the right panel of Fig. 1b). We followed the same procedure in the control condition (i.e., Correct Information Condition), but participants were informed that the hand would be displaced temporarily and then relocated to the same position. After the verbal manipulation, the virtual body was again observed for 60 s, and the two measures (i.e., Body Localization Task first and then the Embodiment Scale) were taken again (T1).

Finally, participants were exposed to the vRHI. During the illusion, participants received a visuo-tactile stimulation: they observed a virtual stick touching the left hand of the avatar while simultaneously receiving tactile stimulation at the same position on their hand (Fig. 1a). The movements of the virtual stick were mapped to the left controller movements of the HMD, and the tactile stimulation was delivered via a stick connected to the controller. The visuo-tactile stimulation lasted 120 s at a frequency of 1 Hz. Following this period, a virtual knife appeared and stabbed the virtual hand. Afterwards, the Body Localization Task and the Embodiment Scale were administered again (T2), following the same order of presentation. Thus, across all phases (T0–T2) and conditions (Misleading and Correct Information), the virtual hand was consistently displayed at a fixed rightward position relative to the physical hand, which likewise remained in the same location (i.e., there was always a mismatch between real and virtual hand positions). The verbal manipulation altered only the instructional content, informing participants of a rightward shift of their hand in the Misleading condition, with no actual change to either the virtual or the physical hand position. Throughout the procedure, we collected SCL to assess the sympathetic response to the virtual threat. See Fig. 1a for the experiment timeline.

Each participant performed a total of six assessments, differing in T1 according to the type of verbal manipulation performed (i.e., Correct or Misleading Information). The experiment resulted in a 3(Time)×2(Verbal Manipulation) factorial within-subject design.

#### SCL apparatus and data pre-processing

The module GSR100c of a Biopac System MP150 (Goleta, USA) was used to record SCL. We set the gain parameter at five mmho/V, and the signal was recorded at a sampling frequency of 100 Hz. The signal was acquired using two electrodes for electrodermal activity measurements (EL507A) positioned on the right hand’s first phalanx of the middle and ring fingers. Highly conductive electrolyte gel was applied to the electrodes to improve the signal-to-noise ratio.

When the virtual knife appeared to the participants after the visuo-tactile stimulation, a trigger indicating the onset of the stimulus was manually recorded using the computer keyboard.

At the end of the recording, we set offline a smoothing factor of 25 (i.e., one-fourth of the sampling rate). Then, we applied a digital high-pass filter at 0.05 HZ to extract phasic Skin Conductance Response (SCR) from the SCL (Andreassi, 2010; Romano et al., 2014). We defined responses with a minimum SCR amplitude of 0.02 µS; no additional relative-amplitude rejection was applied (reject SCRs under: 0% of max). For each condition, we computed the peak-to-peak (P-P) measure elicited by the virtual threat within a time window of 13 s, starting from the appearance of the virtual knife. The duration of the time window was determined by considering the time between the appearance of the knife and the stabbing (approximately 1.5 s), the time required for the SCR to begin after the stimulus (estimated in one to three seconds), the time until the response reached its peak (up to five seconds), and response depletion (Boucsein et al., 2012). To limit spontaneous, stimulus-unrelated SCRs, we applied a 5-s pre-event baseline, computed phasic EDA using a 0.05 Hz high-pass filter, and required a minimum SCR amplitude of 0.02 µS for a response to be considered.

#### Body localization task

During the Body Localization Task, the virtual hand was hidden by a black virtual plane. Participants had to indicate the perceived location of the index fingertip of their actual left hand through a virtual ray cast controlled by the right-hand controller. They were instructed to execute the task ten times as accurately as possible, performing ballistic movements with their right hand starting from a resting position on the table.

From the Body Localization Task, we computed two indices: the classical Proprioceptive Drift and the Virtual Drift. The Proprioceptive Drift reflects the difference between the actual hand position and the estimated position, with a negative value indicating a rightward shift of the perceived positions and a positive value indicating a leftward shift.

The Virtual Drift represents both the deviation from the avatar’s position (i.e., the difference between the virtual hand position and the estimated position) and the precision of the prediction, as it also includes the standard error. For this index, lower scores denote a greater drift towards the virtual hand position or higher estimation precision.

#### Embodiment questionnaire

We measured feelings of ownership for the virtual body and disownership for the real body through the Embodiment Scale (adapted from Romano et al., 2021). The questionnaire included 18 questions rated on a seven-point Likert scale (−3 to + 3). Ten questions assess the embodiment feeling, six capture the disembodiment experience, and two focus on physical sensations. We averaged participants’ responses within each dimension to examine the emergence of the embodiment and disembodiment sensations across conditions. See Supplemental Information for the detailed questionnaire (Table SI1).

### Data analysis

We conducted a series of Bayesian Regression models following a factorial 2(Verbal manipulation)×3(Time) within-subject design. Analyses were conducted with R 4.3.2 (R Core Team, 2023) using the *brms* package (Bürkner, 2017, 2018, 2021).

First, we checked for the presence of outliers and determined the type of distribution for each dependent variable to establish the appropriate distribution family. Weakly informative priors centred around 0 with a standard deviation of 0.5 were applied to regression coefficients, according to those utilized for ANOVA in JASP Software, improving replicability and reproducibility (Wetzels et al., 2012; Rouder et al., 2017). All factors were contrast-coded so that each level was compared with the subsequent level, and the intercept corresponded to the grand mean (i.e., sequential contrast). After performing the posterior predictive check, we interpreted the results by considering the 95% Credible Intervals (CIs). Also, to check our predictions directly, we computed the Bayes Factor to quantify evidence supporting the complete model compared to a model without the predicted effect. Bayesian analysis provides evidence supporting either the null or alternative hypotheses. Finally, to verify the stability of our results, we also performed a robustness check for each analysis, comparing our results with both more (standard deviation = 0.3) and less (standard deviation = 1) informative priors. See Supplemental Information for detailed information regarding priors, posterior predictive check, and robustness check (Sections SI1 and SI2).

#### Embodiment questionnaire

We ran a within-subjects 2 × 3 Bayesian regression for the embodiment and disembodiment factors to assess the subjective aspect of embodiment and disembodiment sensations. We considered as fixed factors Verbal Manipulation (Misleading Information and Correct Information Conditions) and Time (T0, T1, and T2) and their interactions. Participants were set as a cluster variable to account for inter-subject variability and random intercepts and slopes were estimated for the main effects of Verbal Manipulation and Time. Regarding priors specification, regression coefficients were assigned a Normal distribution centered at 0 with a standard deviation of 0.5. For random effects, we allowed for greater variability by setting a larger standard deviation of 1. For the model intercept, we used a prior also centered at zero with a standard deviation of 0.5 but constrained it within the plausible range of −3 to 3, in line with the questionnaire scale. Results were interpreted by examining 95% CIs. Finally, we conducted a directional Bayesian one-sample t-test with a Cauchy prior distribution (*r* = 0.707) to assess whether mere visual exposure already elicits embodiment at T0 (H₁: µ > 0; for details see Supplementary Information, Section SI3).

#### SCR

A Bayesian t-test was performed on the SCR-PP index to compare the arousal response to the virtual threat between the two verbal manipulation conditions (Misleading Information and Correct Information Conditions). In addition to the Bayes Factor, we also extracted the posterior distribution of the standardized paired difference (delta), allowing a clearer interpretation of the difference between conditions. Considering priors specification, a Cauchy prior distribution centred at zero with a scale parameter of √2⁄2 (= 0.707) was applied. We then performed a robustness check with different priors (scale of 1 and 0.3) to verify the stability of the result.

#### Body localization task

The Body Localization Task results were considered computing both the classical Proprioceptive Drift and the novel Virtual Drift score at each time point (T0, T1, and T2) and for each condition (Misleading Information and Correct Information Conditions). First, improbable responses (< −100) were removed, and the presence of outliers in each subject and condition was checked using the boxplot method (*rstatix* package in the R function *identify_outliers*). We considered a percentage of 50% missing values in each condition as a threshold to remove participants. However, no participants presented more than 50% outliers within the same condition, so we removed specific outlier responses but no participants. A total of 264 responses were removed (i.e., 5.51% of the total sample). Also, we tested whether mere visual exposure produces a rightward shift at T0 of the perceived hand position using a directional Bayesian one-sample t-test with a Cauchy prior (*r* = 0.707; H₁: µ < 0; for details see Supplementary Information, Section SI3).

A 2 × 3 Bayesian regression was performed on Proprioceptive Drift to assess the influence of the illusion on the body’s spatial prediction. We considered as fixed factors Verbal Manipulation (Misleading Information and Correct Information Conditions) and Time (T0, T1, and T2) and their interactions. Participants were set as a cluster variable to account for inter-subject variability, and random intercepts and slopes were estimated for the main effects of Verbal Manipulation and Time. Regarding priors specification, regression coefficients and the model intercept were assigned a Normal distribution centered at 0 with a standard deviation of 0.5. For random effects, we allowed for greater variability by setting a larger standard deviation of 1. Results were interpreted by examining 95% CIs.

Virtual drift was computed by subtracting the estimated position from the virtual hand position (considering the average of the absolute value of all responses from each participant in each condition) and adding the standard error. We conducted two associations analyses to investigate the influence of Misleading Information on the precision of the hand’s position prediction. In a Bayesian perspective, T1 (following verbal information and visual exposure) can be treated as an informative prior for T2 (after visuo-tactile stimulation). The Misleading instruction is expected to shift this prior toward the virtual hand, making it more probable that the perceived hand location is closer to the avatar and more precise at T2, with a concomitant increase in ownership. As a prerequisite, we first tested whether virtual drift at T1 predicted drift at T2, after controlling for baseline drift at T0, using a Bayesian regression analysis separately for each condition (Misleading Information and Correct Information Conditions). To quantify the strength of evidence for including T1 as a predictor, we computed BF by comparing the full model (T1 + T0) against a reduced model including only T0. Moreover, to assess the relation between virtual drift in T1 and the embodiment score in T2, we performed a Bayesian regression for both conditions. To quantify the strength of evidence for virtual drift in T1 as a predictor of embodiment in T2, we computed BF by comparing the model against the intercept-only model. For both analyses, we adopted the default Cauchy prior distribution centred at zero with a scale parameter of √2⁄2 (= 0.707), and we extracted posterior estimates for the model parameters, reporting the mean and 95% credible intervals. We then performed a robustness check with different priors (standard deviation of 1 and 0.3) to verify the stability of the result. Finally, to consider only the precision component of localisation, a Bayesian regression on the standard error of the Virtual Drift, assessing the effects of Condition and Time, is reported in the Supplementary Information (Table SI3; Figure SI1).

Data and analysis code are available on the Open Science Framework (OSF) platform at the following link: https://osf.io/z7nbx/?view_only=92e64fef49a14c9bb96fe8c2b63b96a0.

## Results

### Embodiment questionnaire

**Fig. 2:**
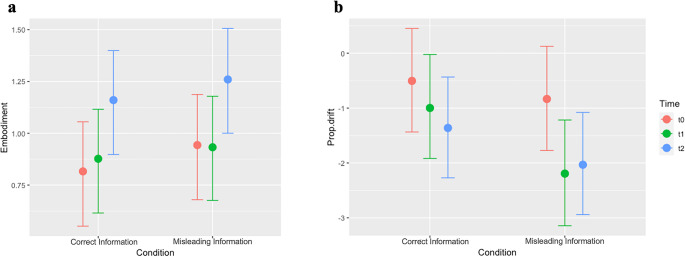
Posterior conditional means from the Bayesian model. Effect of Time and Verbal Manipulation on **(a)** the average scores of the Embodiment factor and **(b)** the proprioceptive drift (real – estimated position). Error bars indicate 95% Credible Interval limits

The Bayesian regression conducted on embodiment scores indicated a main effect of time. As shown in Fig. 2a, the embodiment scores are higher in T2 (i.e., after the vRHI induction) compared to T1 in both conditions (−0.30; 95% CI: [−0.39; −0.22]). It is important to note that the questionnaire scores indicated the presence of embodiment feelings even at T0, following mere visual exposure (values > 0; see also the results of the directional Bayesian one-sample t-test in the Supplementary Information, Section SI3). However, we did not find the expected interaction effect Time $\:\times\:$ Verbal Manipulation (see Table 1 for detailed results). In addition, we found no evidence of a difference between T1 and T0 (Time_T1_: −0.03, 95% CI [− 0.13, 0.06]), nor evidence of a Time_T1_ × Condition_Misleading_ interaction (0.07, 95% CI [− 0.09, 0.24]), indicating that repeated exposure to the virtual hand alone does not enhance embodiment; rather, multisensory stimulation appears necessary to enhance it.

**Table 1:** Results of bayesian regression on embodiment and disembodiment scores

Embodiment effects	Estimate	Est. Error	Lower 95% CI	Upper 95% CI
Intercept	0.99	0.12	0.76	1.23
Time_T1_	−0.03	0.05	−0.13	0.06
Time_T2_	**−0. 30**	**0.05**	**−0.39**	**−0.22**
Condition_Misleading_	0.10	0.05	−0.01	0.20
Time_T1_ *Condition_Misleading_	0.07	0.08	−0.09	0.24
Time _T2_ *Condition_Misleading_	−0.04	0.08	−0.20	0.12
Disembodiment effects	Estimate	Est. Error	Lower 95% CI	Upper 95% CI
Intercept	**−1.27**	**0.11**	**−1.48**	**−1.06**
Time_T1_	0.04	0.06	−0.08	0.16
Time_T2_	−0.03	0.05	−0.13	0.07
Condition_Misleading_	0.01	0.06	−0.10	0.12
Time_T1_ *Condition_Misleading_	−0.10	0.10	−0.30	0.10
Time _T2_ *Condition_Misleading_	0.09	0.10	−0.10	0.28

The table shows the mean (Estimate) and the standard deviation (Est. Error) of the posterior distribution of each effect with the 95% Credible Intervals (lower 95% CI, upper 95% CI). In bold, the posterior distributions without a zero overlapping

Finally, we compared the full model with a model without the expected interaction and computed the BF to test evidence in support of including it. The comparison between models indicates that data are better explained when the interaction Time $\:\times\:$ Verbal Manipulation is not included (BF_incl_ = 0.043). Robustness check confirmed this conclusion across different priors, with BF_incl_ = 0.009 for a less informative prior (standard deviation = 1) and BF_incl_ = 0.061 for a more informative prior (standard deviation = 0.3). See Supplementary Information for the detailed results (Section SI2, Table A).

Contrary to our expectation, Bayesian regression on the disembodiment scores revealed no changes across Time and Verbal Manipulation (see Table 1 for detailed results). The comparison between models indicates that data are better explained when the interaction Time $\:\times\:$ Verbal Manipulation is not included (BF_incl_ = 0.04). Robustness checks confirmed this conclusion across different priors, with BF_incl_ = 0.001 for a less informative prior (standard deviation = 1) and BF_incl_ = 0.011 for a more informative prior (standard deviation = 0.3). See Supplementary Information for the detailed results (Section SI2, Table B).

Results for the physical sensations factor are reported in the Supplemental Information (Table SI2), as this factor is also included in the Embodiment Scale, but it is outside the focus of the present study.

### SCR

The Bayesian t-test indicated moderate evidence in favour of the absence of differences in the SCR P-P between Verbal Manipulation Conditions (BF_01_ = 4.82 ± 0.08%). This result is further supported by the posterior distribution of the standardized paired difference between the Misleading Information and Correct Information Conditions (−0.11; 95% CI [−0.34, 0.11]). This result suggests that a similar arousal is induced by a threat to the virtual hand, regardless of the type of verbal instructions participants received. Robustness checks confirmed the result: evidence in favor of the null model remained stable across different prior specifications, with anecdotal evidence (BF₀₁ = 2.39) for a more informative prior (standard deviation = 0.3) and moderate evidence (BF₀₁ = 6.66) for a less informative prior (standard deviation = 1.0). See Supplementary Information for more details (Section SI2, Table C).

### Proprioceptive drift

The Bayesian regression on the Proprioceptive Drift revealed an interaction effect between Verbal Manipulation and Time (Fig. 2b). Specifically, when comparing T0 and T1, we found a higher proprioceptive drift towards the right (i.e., towards the virtual hand) after the Misleading Information Condition than after the Control Condition (0.85; 95% CIs: [0.42; 1.27]). This result confirmed our first hypothesis: a pronounced shift of the perceived hand position towards the position suggested during the Misleading Information Condition was observed. Interestingly, the directional Bayesian one-sample t-test (see Supplementary Information, Section SI3) showed that already at T0 the mean drift was negative, consistent with a rightward shift toward the virtual hand following mere visual exposure. Contrary to our second hypothesis, when comparing T1 and T2, the proprioceptive drift towards the right was lower after the misleading information than after the correct instructions (−0.46; 95% CIs: [−0.88; −0.04]). Thus, the multisensory stimulation during the vRHI appears to affect the perceived hand position only in the control and not in the Misleading Information Condition. We also found significant main effects of Verbal Manipulation (Misleading vs. Correct Information = −0.73; 95% CI: [−1.39; −0.07]) and time (T1 vs. T0 = 0.92; 95% CI: [0.30; 1.51]). See Table 2 for detailed results.

As predicted, the BF obtained for the inclusion of the Time $\:\times\:\:$Verbal Manipulation interaction, calculated by comparing the full model with a model without the expected interaction effect, demonstrates that data are better explained when the interaction Time $\:\times\:\:$Verbal Manipulation is included (BF_incl_ = 351.12). Robustness checks confirmed this conclusion across different priors, with BF_incl_ = 777.487 for a less informative prior (standard deviation = 1) and BF_incl_ = 74.538 for a more informative prior (standard deviation = 0.3). See Supplementary Information for the detailed results (Section SI2, Table D).

**Table 2:** Results of Bayesian regression on Proprioceptive Drift.

Embodiment effects	Estimate	Est. Error	Lower 95% CI	Upper 95% CI
Intercept	**−1.35**	**0.41**	**−2.15**	**−0.53**
Time_T1_	**0.92**	**0.30**	**0.30**	**1.51**
Time_T2_	0.14	0.27	−0.38	0.67
Condition_Misleading_	**−0.73**	**0.34**	**−1.39**	**−0.07**
Time_T1_ *Condition_Misleading_	**0.85**	**0.22**	**0.42**	**1.27**
Time _T2_ *Condition_Misleading_	**−0.46**	**0.21**	**−0.88**	**−0.04**

The table shows the mean (Estimate) and the standard deviation (Est.Error) of the posterior distribution of each effect with the 95% Credible Intervals (lower 95% CI, upper 95% CI). In bold, the posterior distributions without a zero overlapping

### Virtual drift

**Fig. 3:**
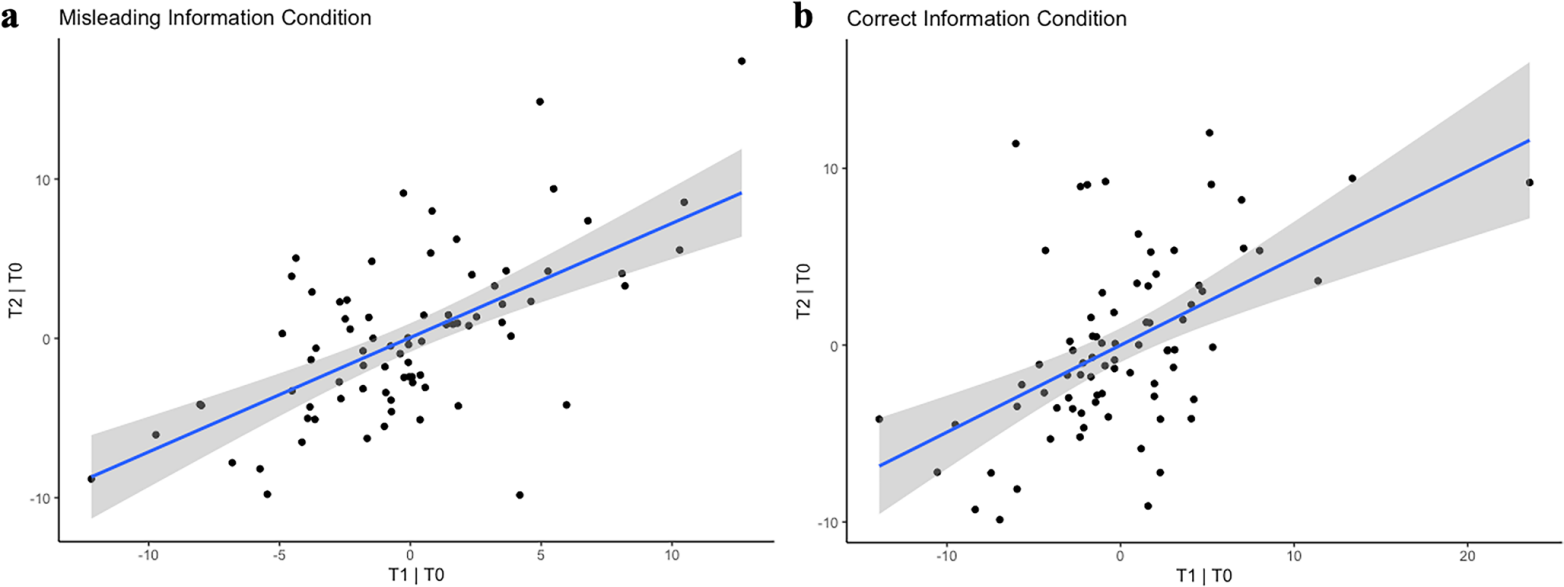
Relation between virtual drift at T1 and virtual drift at t2, net of T0. Evidence of a positive relation between the virtual drift in T1 and T2 while controlling for virtual drift in T0 in both Misleading Information **(a)** and Correct Information **(b)** conditions

For the Misleading Information Condition, the Bayesian linear regression analysis to assess whether virtual drift at T1 predicted drift at T2, after controlling for baseline drift at T0, provides evidence of the effect (0.71; 95% CI[0.51, 0.92]). The result is also confirmed by the comparison between the full and reduced models, which provides strong evidence that virtual drift at T1 enhances prediction of T2 scores, regardless of baseline levels (BF = 9.21 × 10⁶). Similarly, for the Control condition, results provide strong evidence that virtual drift at T1 predicted T2 scores, after controlling for baseline drift (0.48; 95% CI [0.30, 0.67]), and the comparison between models supported the finding (BF = 1.4 × 10^4^). Thus, for both conditions, results indicated strong evidence in favor of including T1 as a predictor of T2, net of T0 (see Fig. 3). The robustness check with less (standard deviation = 1) and more informative (standard deviation = 0.3) priors supported the result. See Supplementary Information for more details (Section SI2, Table E).

Considering the Bayesian regression to assess the relation between virtual drift in T1 and the embodiment score in T2, results indicate that virtual drift at T1 does not predict embodiment at T2 in the Misleading Information Condition (−0.001; 95% CI [−0.032, 0.030]), suggesting a lack of association between the two variables. Also, we found moderate evidence against the inclusion of T1 virtual drift as a predictor of embodiment at T2 (BF₀₁ = 4.30). In the same way, for the Control condition, results indicate that virtual drift at T1 does not appear to be a predictor of embodiment at T2 (− 0.016; 95% CI [− 0.043, 0.009]) and we found anecdotal evidence against the inclusion of T1 virtual drift in the model (BF₀₁ = 2.01). The robustness checks confirmed the results: evidence supporting the absence of a relationship between virtual drift at T1 and embodiment at T2 remained consistent across different prior specifications in both conditions. See Supplementary Information for more details(Section SI2, Table F).

## Discussion

In the present study, we investigated how altering predictions about the hand’s location impacts the emergence of body ownership sensations. We hypothesized that body ownership and spatial representation are so closely associated that body ownership is causally linked to the prediction of the body’s spatial position. By employing verbal manipulation, we aimed to manipulate the predicted position of the body in space, examining the subsequent effects on the perceived body’s position and ownership and disownership sensations. Participants were exposed to a RHI in virtual reality. Changes in body ownership and the perceived position of the body in space were assessed at three time-points: after visual exposure to the virtual body (T0), after verbal manipulation (T1), and after 120 s of synchronous visuotactile stimulation for vRHI induction (T2). Results revealed that type of verbal instructions influences predictions of body location in space but not the emergence of ownership feelings. In contrast, multisensory body illusions affect both body ownership sensations and the perceived spatial position of the body.

Specifically, we analyzed the shift in perceived body position by considering changes in the proprioceptive drift (i.e., the difference between actual and estimated hand positions) across conditions and time points. As expected, participants reported the hand position as located further to the right after the misleading verbal information, aligning with the direction indicated during the verbal manipulation. This result indicated that verbal cues regarding a rightward hand shift altered the predictions about the hand’s position despite the hand, after being temporally moved, returned to its original position. Classically, proprioceptive drift has been explained as resulting from integrating different sensory information (Abdulkarim & Ehrsson, 2016; Botvinick & Cohen, 1998; Litwin, 2020; Makin et al., 2008). Our results also demonstrated that top-down mechanisms can substantially modulate predictions about body location in space, as verbal manipulation can effectively alter the perceived body position, even when the actual position remains unchanged. Thus, proprioceptive drift may not be solely driven by bottom-up multisensory integration but can also be shaped by participants’ beliefs and expectations.

However, the multisensory body illusion induced after the Misleading Information Condition did not shift the perceived hand’s position further. Previous works have shown the effect of the RHI in inducing a change in the perceived hand location toward the rubber hand (Longo et al., 2008; Romano et al., 2021). In this study, we observed this effect only in the control condition, in which the perceived hand position is shifted to the right (i.e., toward the virtual hand location) after the vRHI induction. Contrary to our expectations, it seems that the synchronous visuo-tactile stimulation fails to induce additional shifts in perceived body position after the Misleading Information Condition, consistent with a ceiling effect. Assuming a limited plausible range for predicted hand position, the verbal manipulation appears to have saturated the rightward shift, thereby preventing further change after the vRHI.

If predictions of body location in space appear modulated by verbal manipulation, the emergence of embodiment feelings would instead be more related to multisensory mechanisms. Contrary to our hypothesis, verbal manipulation did not increase ownership sensations. Thus, perceiving one’s own hand as closer to the virtual one following the verbal manipulation does not seem to enhance ownership feelings toward the virtual hand. Ownership feelings were indeed increased only after the vRHI in both conditions due to the synchronous and repetitive congruency between visual and tactile stimuli, supporting previous results about the RHI in virtual reality (e.g., Slater et al., 2008; Pyasik et al., 2020). Particularly, we found an increase in embodiment sensation compared to a condition of mere observation of the virtual body instead of the classical control of a multisensory asynchronous stimulation (Botvinick & Cohen, 1998; Slater et al., 2008). Our findings corroborate previous works indicating that even mere visual exposure to a virtual body can evoke a sense of body ownership (Frisco et al., 2024; Lenggenhager et al., 2007; Tieri et al., 2015). However, the multisensory information coming from the body and the avatar increased the sensation of owning the avatar’s hand, supporting previous findings (Frisco et al., 2024; Longo et al., 2008; Romano et al., 2021; Slater et al., 2008). Our results suggest that verbal cues do not influence ownership sensation, which is related to multisensory mechanisms modulated by the vRHI.

To address specifically the relationship between the emergence of embodiment sensations and the prediction of the body’s position, we examined the relation between the virtual drift after verbal manipulation (T1) and both the virtual drift and the embodiment score after the vRHI (T2). Results revealed that the predicted location in T1 influenced the spatial prediction but not the embodiment sensations after the illusion. This result would indicate that while there is an association between the prediction of the body position and subsequent spatial predictions, this connection does not extend to the subjective experience of embodiment. Consequently, our hypothesis of a causal link between body ownership and spatial body predictions may not be fully supported.

Our findings may indicate that a change in perceived hand position may be necessary to elicit embodiment feelings but is not sufficient because the recalibration of the perceived hand position occurs independently of the change in ownership feelings. Indeed, we consistently observe an increase in embodiment feelings when there is also a shift in the perceived hand position, and we do not encounter any condition where embodiment is present without such a shift. Conversely, body spatial displacement occurs even without a change in embodiment response (i.e., Misleading Information Condition), confirming previous works (Holmes et al., 2006; Makin et al., 2008), showing that proprioceptive drift and body ownership are partially dissociable. This evidence is further supported, in a broader sense, by studies on tool use, where participants update the implicit morphological representation of their arm without any corresponding modulation of body ownership (e.g., Cardinali et al., 2009; Canzoneri et al., 2013; D’Angelo et al., 2018).

This interpretation aligned also with prior observations in the context of the embodiment phenomena, particularly regarding the relationship between embodiment sensations and the referral of touch (i.e., the sensation of touch on one’s hand is perceived as originating from the location of the visual stimulus on the rubber hand). A recent work suggested that the referral of touch and the embodiment may represent different facets of the illusion, with the referral of touch potentially preceding the sense of ownership as a determinant of its manifestation (Tosi et al., 2024). Similarly, the recalibration of proprioceptive coordinates may be a prerequisite for inducing embodiment; however, future research is needed to verify whether embodiment sensations can be induced without a perceived body spatial displacement. An alternative interpretation is that the greater displacement in the Misleading condition reflects evidence accumulation from convergent visual and verbal cues. In our design, the visual input was constant across conditions, and what varied was the verbal information, which was either congruent (Misleading) or incongruent (Correct) with the real hand position. Stronger updating in the Misleading condition would thus follow from cue convergence, rather than (or in addition to) a change driven solely by prior spatial expectations.

Our initial hypothesis, supported by previous studies, reported the emergence of disownership feelings during the RHI, where misalignment between the real and fake body is present (Longo et al., 2008; Romano et al., 2021). However, in this study, we found that disembodiment sensations did not vary across different time points and conditions, consistent with the results of our previous work (Frisco et al., 2024). In our prior study, participants experienced the vRHI in two possible location conditions: an aligned condition, where the virtual hand position matched the real hand position, and a misaligned condition, where the virtual hand was shifted to the right, closer to the body midline. Disembodiment sensations before and after the vRHI induction in these two conditions showed no changes in disownership feelings. However, a potential confound emerged by debriefing participants at the end of the experiment. Indeed, when the misaligned condition was presented first, participants perceived the real and the virtual hand as located in the same position in both the aligned and misaligned conditions. Conversely, participants accurately distinguished the two different spatial locations when the aligned condition was presented first. Thus, the same mechanism may explain the present finding. Considering the participants’ responses to Question 6 of the Embodiment Scale, which specifically addressed the perceived position of the real hand relative to the virtual hand (“*It seemed like my hand was in the location where the virtual hand was”)*, participants consistently agreed with this statement, suggesting that they perceived their hand to be located approximately in the same location as the virtual one (i.e., closer to the body; see Supplementary Information for descriptives regarding Question 6; Table SI4 and Figure SI2). It is possible that, during the RHI in a virtual reality setup, visual capture may mask spatial discrepancies. Since the virtual hand is shifted from the real hand’s location, the strong visual capture in the virtual reality environment may result in a larger perceived space for the hand’s location, leading to a broader space of embodiment. Consequently, disownership sensations might not emerge, as participants may not explicitly recognize the spatial mismatch between the virtual and real hand positions.

The main limitation of the present study is that our hypothesis has been developed within the specific framework of the virtual RHI, even though findings from the previous work found a link between body spatial predictions and embodiment feelings, specifically in the 1pp-FBI (Frisco et al., 2024). As discussed, a confound emerged in both the present and previous work, related to participants’ difficulty in consistently detecting the misaligned position of the virtual relative to the real hand in the virtual reality environment. This confound may have influenced the results. Therefore, future studies are needed to replicate and extend the present findings in the context of other bodily illusions. Moreover, an additional condition with a verbal manipulation opposite to the canonical RHI displacement could be useful to better control the effects. Such control would clarify whether verbal manipulation can shift localisation even against the spatial bias induced by the observation of the virtual hand.

## Conclusion

In conclusion, our study showed distinct effects of verbal manipulation and the multisensory body illusion. Specifically, verbal manipulation influenced the prediction of the body’s position in space, whereas the multisensory illusion modulated both embodiment sensations and perceived spatial position. The perception of one’s body in space could be related to both multisensory integration and more cognitive and top-down mechanisms (i.e., verbal instructions). Our results partially confirmed our initial hypotheses and may indicate that the body’s spatial displacement is necessary to evoke embodiment sensations but not sufficient, as body position recalibration can occur independently of changes in embodiment feelings.

## Significance Statement

Our study reveals that how we perceive our body’s position in space depends on both spatial predictions and multisensory integration between vision and touch. While spatial predictions can bias perceived location, they do not affect the feeling that the body belongs to oneself. These findings refine our understanding of bodily self-awareness and may inform applications of virtual reality and neurorehabilitation therapies targeting body perception and ownership disturbances.

## Supplementary Information

Below is the link to the electronic supplementary material.

Supplementary Material 1 (DOCX 1.96 MB)

## Data Availability

Data of the experiments are available at the OSF at the following link: [https://osf.io/z7nbx/?view_only=92e64fef49a14c9bb96fe8c2b63b96a0](https:/osf.io/z7nbx/?view_only=92e64fef49a14c9bb96fe8c2b63b96a0).

## Code Availability

Analysis scripts of the experiments are available at the OSF at the following link:

https://osf.io/z7nbx/?view_only=92e64fef49a14c9bb96fe8c2b63b96a0.

## References

[CR1] Abdulkarim, Z., & Ehrsson, H. H. (2016). No causal link between changes in hand position sense and feeling of limb ownership in the rubber hand illusion. *Attention, Perception & Psychophysics,**78*(2), 707–720. 10.3758/s13414-015-1016-0 PMC4744264 26555651

[CR2] Andreassi, J. L. (2010). *Psychophysiology: Human behavior and physiological response*. Psychology press.

[CR3] Blanke, O. (2012). Multisensory brain mechanisms of bodily self-consciousness. *Nature Reviews Neuroscience*, *13*(8), 556–571. 22805909 10.1038/nrn3292

[CR4] Botvinick, M., & Cohen, J. (1998). Rubber hands ‘feel’touch that eyes see. *Nature*, *391*(6669), 756–756. 9486643 10.1038/35784

[CR5] Boucsein, W., Fowles, D. C., Grimnes, S., Ben-Shakhar, G., Roth, W. T., Dawson, M. E., & Filion, D. L. (2012). Publication recommendations for electrodermal measurements. *Psychophysiology,**49*(8), 1017–1034. 10.1111/j.1469-8986.2012.01384.x 22680988 10.1111/j.1469-8986.2012.01384.x

[CR35] Bürkner, P. (2017). brms: An R package for bayesian multilevel models using stan. *Journal of Statistical Software*, *80*(1), 1–28. 10.18637/jss.v080.i01

[CR36] Bürkner, P. (2018). Advanced bayesian multilevel modeling with the R Package brms. *The R Journal*, *10*(1), 395–411. 10.32614/RJ-2018-017

[CR37] Bürkner, P. (2021). Bayesian item response modeling in R with brms and Stan. *Journal of Statistical Software*, *100*(5), 1–54. 10.18637/jss.v100.i05

[CR6] Canzoneri, E., Ubaldi, S., Rastelli, V., Finisguerra, A., Bassolino, M., & Serino, A. (2013). Tool-use reshapes the boundaries of body and peripersonal space representations. *Experimental Brain Research,**228*(1), 25–42. 23640106 10.1007/s00221-013-3532-2

[CR7] Cardinali, L., Frassinetti, F., Brozzoli, C., Urquizar, C., Roy, A. C., & Farnè, A. (2009). Tool-use induces morphological updating of the body schema. *Current Biology,**19*(12), R478–R479. 19549491 10.1016/j.cub.2009.05.009

[CR8] D’Angelo, M., Di Pellegrino, G., Seriani, S., Gallina, P., & Frassinetti, F. (2018). The sense of agency shapes body schema and peripersonal space. *Scientific Reports,**8*(1), Article 13847. 30218103 10.1038/s41598-018-32238-z PMC6138644

[CR9] Ehrsson, H. H. (2019). Multisensory processes in body ownership. *Multisensory Perception: From Laboratory to Clinic* (pp. 179–200). Academic Press.

[CR10] Frisco, F., Bruno, V., Romano, D., & Tosi, G. (2024). I am where I believe my body is: The interplay between body spatial prediction and body ownership. *PLoS One, 19*(12), e0314271*.* 39666650 10.1371/journal.pone.0314271 PMC11637335

[CR11] Holmes, N. P., Snijders, H. J., & Spence, C. (2006). Reaching with alien limbs: Visual exposure to prosthetic hands in a mirror biases proprioception without accompanying illusions of ownership. *Perception & Psychophysics,**68*, 685–701. 16933431 10.3758/bf03208768 PMC1564193

[CR12] Kalckert, A., & Ehrsson, H. H. (2012). Moving a rubber hand that feels like your own: A dissociation of ownership and agency. *Frontiers in Human Neuroscience*, *6*, 1–14. 10.3389/fnhum.2012.00040 22435056 10.3389/fnhum.2012.00040 PMC3303087

[CR13] Lenggenhager, B., Tadi, T., Metzinger, T., & Blanke, O. (2007). Video ergo sum: Manipulating bodily self-consciousness. *Science,**317*(5841), 1096–1099. 17717189 10.1126/science.1143439

[CR14] Lira, M., Egito, J. H., Dall’Agnol, P. A., Amodio, D. M., Gonçalves, Ó. F., & Boggio, P. S. (2017). The influence of skin colour on the experience of ownership in the rub- ber hand illusion. *Scientific Reports*, *7*(1), 1–13. 10.1038/s41598-017-16137-3 29147006 10.1038/s41598-017-16137-3 PMC5691152

[CR38] Litwin, P., Zybura, B., & Motyka, P. (2020). Tactile information counteracts the attenuation of rubber hand illusion attributable to increased visuo-proprioceptive divergence. *PLoS One*, *15*(12), e0244594. 10.1371/journal.pone.0244594 PMC7773248 33378385

[CR15] Lloyd, D. M. (2007). Spatial limits on referred touch to an alien limb may reflect boundaries of visuo-tactile peripersonal space surrounding the hand. *Brain and Cognition,**64*(1), 104–109. 10.1016/j.bandc.2006.09.013 17118503 10.1016/j.bandc.2006.09.013

[CR16] Longo, M. R., Schüür, F., Kammers, M. P., Tsakiris, M., & Haggard, P. (2008). What is embodiment? A psychometric approach. *Cognition,**107*(3), 978–998. 18262508 10.1016/j.cognition.2007.12.004

[CR17] Longo, M. R., Azañón, E., & Haggard, P. (2010). More than skin deep: Body representation beyond primary somatosensory cortex. *Neuropsychologia,**48*, 655–668. 10.1016/j.neuropsychologia.2009.08.022 19720070 10.1016/j.neuropsychologia.2009.08.022

[CR18] Makin, T. R., Holmes, N. P., & Ehrsson, H. H. (2008). On the other hand: Dummy hands and peripersonal space. *Behavioural Brain Research,**191*(1), 1–10. 18423906 10.1016/j.bbr.2008.02.041

[CR19] Perugini, M., Gallucci, M., & Costantini, G. (2014). Safeguard power as a protection against imprecise power estimates. *Perspectives on Psychological Science,**9*(3), Article 1745691614528519. 10.1177/1745691614528519 10.1177/1745691614528519 26173267

[CR20] Pyasik, M., Tieri, G., & Pia, L. (2020). Visual appearance of the virtual hand affects embodiment in the virtual hand illusion. *Scientific Reports,**10*(1), Article 5412. 32214171 10.1038/s41598-020-62394-0 PMC7096421

[CR21] R Core Team. (2023). *R: A Language and environment for statistical computing*. R Foundation for Statistical computing.

[CR22] Rohde, M., Luca, M., & Ernst, M. O. (2011). The rubber hand illusion: Feeling of ownership and proprioceptive drift Do not go hand in hand. *PLoS One,**6*, Article e21659. 10.1371/journal.pone.0021659 21738756 10.1371/journal.pone.0021659 PMC3125296

[CR23] Romano, D., Maravita, A., & Perugini, M. (2021). Psychometric properties of the embodiment scale for the rubber hand illusion and its relation with individual differences. *Scientific Reports,**11*(1), Article 5029. 33658576 10.1038/s41598-021-84595-x PMC7930179

[CR24] Romano, D., Pfeiffer, C., Maravita, A., & Blanke, O. (2014). Illusory self-identification with an avatar reduces arousal responses to painful stimuli. *Behavioural Brain Research,**261*, 275–281. 24412686 10.1016/j.bbr.2013.12.049

[CR25] Rouder, J. N., Morey, R. D., Verhagen, J., Swagman, A. R., & Wagenmakers, E. J. (2017). Bayesian analysis of factorial designs. *Psychological Methods,**22*(2), 304. 27280448 10.1037/met0000057

[CR26] Serino, A., Alsmith, A., Costantini, M., Mandrigin, A., Tajadura-Jimenez, A., & Lopez, C. (2013). Bodily ownership and self-location: Components of bodily self-consciousness. *Consciousness and Cognition,**22*(4), 1239–1252. 24025475 10.1016/j.concog.2013.08.013

[CR27] Slater, M., Pérez Marcos, D., Ehrsson, H., & Sanchez-Vives, M. V. (2008). Towards a digital body: The virtual arm illusion. *Frontiers in Human Neuroscience,**2*, Article 181. 10.3389/neuro.09.006.2008 PMC2572198 18958207

[CR28] Tieri, G., Tidoni, E., Pavone, E. F., Aglioti, S. M., & Moro, V. (2015). Mere observation of body discontinuity affects perceived ownership and vicarious agency over a virtual hand. *Experimental Brain Research,**233*, 1247–1259. 25618006 10.1007/s00221-015-4202-3

[CR29] Tosi, G., Kalckert, A., Sivasubramanian, A. K., & Romano, D. (2024). The rubber hand illusion questionnaire: An exploratory graph analysis of ownership, referral of touch, and control statements. *Attention, Perception & Psychophysics, 86*(8), 2866–2876. 10.3758/s13414-024-02964-w 10.3758/s13414-024-02964-w PMC11652674 39349922

[CR30] Tosi, G., Maravita, A., & Romano, D. (2022). I am the metre: The representation of one’s body size affects the perception of tactile distances on the body. *Quarterly Journal of Experimental Psychology*, *75*(4), 583–597. 10.1177/17470218211044488 34427459

[CR31] Tosi, G., Mentesana, B., & Romano, D. (2023). The correlation between proprioceptive drift and subjective embodiment during the rubber hand illusion: A meta-analytic approach. *Quarterly Journal of Experimental Psychology*, *76*(10), 2197–2207. 10.1177/17470218231156849 36880657

[CR32] Tosi, G., & Romano, D. (2023). The network of the subjective experience in embodiment phenomena. *Psychological Research = Psychologische Forschung,**87*(4), 1043–1056. 35871696 10.1007/s00426-022-01714-7 PMC10191983

[CR33] Wetzels, R., Grasman, R. P., & Wagenmakers, E. J. (2012). A default Bayesian hypothesis test for ANOVA designs. *The American Statistician,**66*(2), 104–111.

[CR34] World Medical Organization. (1996). Declaration of Helsinki. *Br Med J*, *313*, 1448–1449.

