# Supplementary material for: Trust me, you are there: the role of verbal manipulation on embodiment and body localization
Source: Psychol Res. 2026 Apr 11;90(2):73. doi: 10.1007/s00426-026-02271-z (PMC13070051; doi:10.1007/s00426-026-02271-z)
Supplement: Supplementary file 1 — Supplementary Material 1 (DOCX 1.96 MB) [file 426_2026_2271_MOESM1_ESM.docx]

**Supplementary Information: “Trust me, you are there: the role of verbal conditioning on embodiment and body localization”**

**Francesca Frisco^a,b,c^, Vito Bruno^a^, Daniele Romano^a,b,c^ , Giorgia Tosi^a,b,c*^**

*a Department of Psychology, University of Milan-Bicocca. Milan, Italy*

*b Milan Center for Neuroscience. Milan, Italy*

*c* *Mind and Behavior Technological Center, University of Milan-Bicocca. Milan, Italy*

* **Corresponding Author:**

1. Giorgia Tosi, [giorgia.tosi@unimib.it](mailto:giorgia.tosi@unimib.it)

Department of Psychology, University of Milan – Bicocca, Building U6, Room

1724. Piazza dell'Ateneo Nuovo 1, 20126, Milan, Italy. Tel: +39 02 64483866,

ORCID: 0000-0001-6405-3310

**Co-authors:**

1. Francesca Frisco, [francesca.frisco@unimib.it](mailto:francesca.frisco@unimib.it), ORCID: 0000-0002-6167-6587
2. Vito Bruno, [vito.bruno@unimib.it](mailto:vito.bruno@unimib.it)
3. Daniele Romano, [daniele.romano@unimib.it](mailto:daniele.romano@unimib.it), ORCID: 0000-0002-6366-8433

**Supplementary Information Index**

- **Table SI1. Embodiment Scale (ES)**
- **Table SI2. Results of the Bayesian regression on Physical Sensations factor of the Embodiment Scale (ES).**
- **Table SI3. Results of the Bayesian regression on Virtual Drift Standard Error**
- **Figure SI1**
- **Table SI4. Descriptives of responses to Question 6 of the Embodiment Scale**
- **Figure SI2**
- **Section SI1. Models and Posterior Predictive Check for each analysis performed.**
- Bayesian Regression on Embodiment
- Bayesian Regression on Disembodiment
- Bayesian Regression on Physical Sensations
- Bayesian Regression on Proprioceptive Drift
- **Section SI2. Robustness Check**
- Table A. Bayesian regression on Embodiment with less (standard deviation = 1) and more (standard deviation = 0.3) informative priors.
- Table B. Bayesian regression on Disembodiment with less (standard deviation= 1) and more (standard deviation = 0.3) informative priors
- Table C. Bayesian t-test on SCR-PP between with less (standard deviation = 1) and more (standard deviation = 0.3) informative priors.
- Table D. Bayesian regression on Proprioceptive with less (standard deviation = 1) and more (standard deviation = 0.3) informative priors.
- Table E. Effect of Virtual Drift in T1 on Virtual Drift in T2, regardless virtual drift in T0 with less (standard deviation = 1) and more (standard deviation = 0.3) informative priors
- Table F. Effect of Virtual Drift in T1 on Embodiment Score in T2 between with less (standard deviation = 1) and more (standard deviation = 0.3) informative priors.
- **Section SI3. One-Sample t-tests on Embodiment and Proprioceptive Drift at T0**

**Supplementary Materials**

**Table SI1**. **Embodiment Scale (ES)**

| Factor | Item (Factor) | From -3 to +3, how much do you agree with the following statements.  “During the block …” |  |  |  |  |  |  |  |
| --- | --- | --- | --- | --- | --- | --- | --- | --- | --- |
| Embodiment | 1 (E1) | ...it seemed like I was looking directly at my own hand, rather than at a virtual hand | -3 | -2 | -1 | -0 | +1 | +2 | +3 |
|  | 2 (E2) | ...it seemed like the virtual hand began to resemble my real hand | -3 | -2 | -1 | -0 | +1 | +2 | +3 |
|  | 3 (E3) | ...it seemed like the virtual hand belonged to me | -3 | -2 | -1 | -0 | +1 | +2 | +3 |
|  | 4 (E4) | ...it seemed like the virtual hand was my hand | -3 | -2 | -1 | -0 | +1 | +2 | +3 |
|  | 5 (E5) | ...it seemed like the virtual hand was part of my body | -3 | -2 | -1 | -0 | +1 | +2 | +3 |
|  | 6 (E6) | ...it seemed like my hand was in the location where the virtual hand was | -3 | -2 | -1 | -0 | +1 | +2 | +3 |
|  | 7 (E7) | ...it seemed like the virtual handwas in the location where my hand was | -3 | -2 | -1 | -0 | +1 | +2 | +3 |
|  | 8 (E8) | ...it seemed like the touch I felt was caused by the paintbrush touching the virtual hand | -3 | -2 | -1 | -0 | +1 | +2 | +3 |
|  | 9 (E9) | ...it seemed like I could have moved the virtual hand if I had wanted | -3 | -2 | -1 | -0 | +1 | +2 | +3 |
|  | 10 (E10) | ...it seemed like I was in control of the virtual hand | -3 | -2 | -1 | -0 | +1 | +2 | +3 |
| Disembodiment | 11 (D1) | ...it seemed like I was unable to move my hand | -3 | -2 | -1 | -0 | +1 | +2 | +3 |
|  | 12 (D2) | ...it seemed like I couldn’t really tell where my hand was | -3 | -2 | -1 | -0 | +1 | +2 | +3 |
|  | 13 (D3) | ...it seemed like my hand had disappeared | -3 | -2 | -1 | -0 | +1 | +2 | +3 |
|  | 14 (D4) | ...it seemed like my hand was out of my control | -3 | -2 | -1 | -0 | +1 | +2 | +3 |
|  | 15 (D5) | ...it seemed like my hand was moving towards the virtual hand | -3 | -2 | -1 | -0 | +1 | +2 | +3 |
|  | 16 (D6) | ...it seemed like the virtual hand was moving towards my hand | -3 | -2 | -1 | -0 | +1 | +2 | +3 |
| Physical Sensations | 17 (P1_r) | ...the touch of the paintbrush on my hand was pleasant | -3 | -2 | -1 | -0 | +1 | +2 | +3 |
|  | 18 (P2) | ...I had the sensation of pins and needles in my hand | -3 | -2 | -1 | -0 | +1 | +2 | +3 |

Questionnaire’s items used to assess embodiment and disembodiment feelings. Participants rated their agreement on 18 questions on a seven-point Likert scale (-3 to +3). Ten questions aimed to capture the embodiment experience (i.e., from E1 to E10), six focused on disembodiment (i.e., from D1 to D6), and two addressed physical sensations (i.e., P1_r and P2). Items E8 and P1_r were not evaluated during T0 and T1 assessment because participants did not experience any touch during the visual exposure. Item P1_r was reversed before analyses.

**Table SI2. Results of the Bayesian regression on Physical Sensations factor of the Embodiment Scale (ES).**

| Physical Sensations (ES) | Estimate | Est. Error | Lower  95% CI | Upper  95% CI |
| --- | --- | --- | --- | --- |
| *Intercept[1]* | **-1.33** | **0.49** | **-2.32** | **-0.39** |
| *Intercept[2]* | 0.90 | 0.49 | -0.04 | 1.87 |
| *Intercept[3]* | **2.22** | **0.50** | **1.25** | **3.24** |
| *Intercept[4]* | **4.07** | **0.55** | **3.04** | **5.18** |
| *Intercept[5]* | **6.21** | **0.64** | **5.01** | **7.53** |
| *Intercept[6]* | **8.67** | **0.82** | **7.17** | **10.35** |
| *Time_T1_* | -0.35 | 0.29 | -0.92 | 0.20 |
| *Time_T2_* | **-1.79** | **0.51** | **-2.82** | **-0.80** |
| *Condition_Misleading_* | 0.10 | 0.28 | -0.44 | 0.66 |
| *Time_T1_:Condition_Misleading_* | -0.11 | 0.54 | -1.18 | 0.95 |
| *Time_T2_:Condition_Misleading_* | 0.10 | 0.49 | -0.87 | 1.06 |

The table shows the mean (Estimate) and the standard deviation (Est.Error) of the posterior distribution of each effect with the 95% Credible Intervals (lower 95% CI, upper 95% CI). In bold, the posterior distributions without a zero overlapping.

**Table SI3. Results of the Bayesian** **Regression on the Standard Error (SE) of the Virtual Drift**

| **SE_VD Effects** | **Estimate** | **Est. Error** | Lower  95% CI | Upper  95% CI |
| --- | --- | --- | --- | --- |
| Intercept | -0.06 | 0.07 | -0.19 | 0.08 |
| Time_T1_ | -0.10 | 0.07 | -0.24 | 0.05 |
| **Time_T2_** | **-0.20** | **0.07** | **-0.34** | **-0.05** |
| Condition_Misleading_ | -0.08 | 0.07 | -0.22 | 0.07 |
| **Time_T1_*Condition_Misleading_** | **0.06** | **0.10** | **-0.14** | **0.26** |
| **Time _T2_* Condition_Misleading_** | **0.09** | **0.10** | **-0.11** | **0.30** |

The table shows the mean (Estimate) and the standard deviation (Est.Error) of the posterior distribution of each effect with the 95% Credible Intervals (lower 95% CI, upper 95% CI). In bold, the posterior distributions without a zero overlapping.


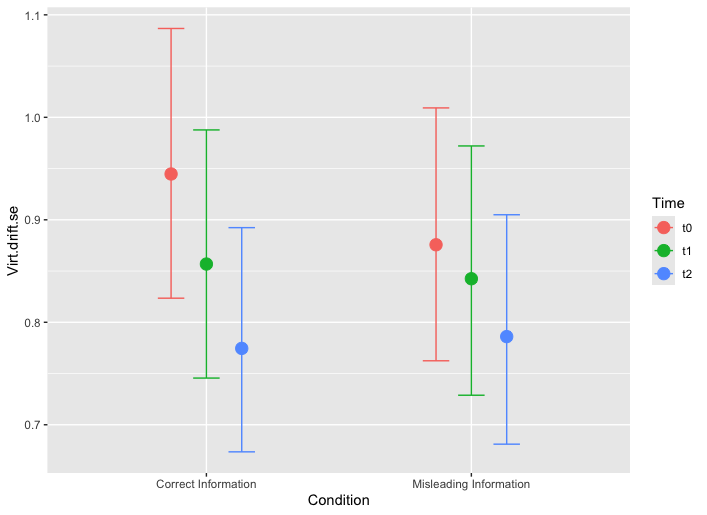


**Figure SI1. Results of the Bayesian Regression on the Standard Error (SE) of the Virtual Drift.** The Bayesian regression on the SE of Virtual Drift showed a main effect of time: the SE is lower in T2 compared to T1 (-0.20; 95% CI: [-0.34; -0.05]). This result indicated that multisensory visuo-tactile stimulation improved localisation precision, regardless of the verbal manipulation conditions.

**Table SI4. Descriptives of responses to Question 6 of the Embodiment Scale**

| *Time* | *Condition* | *N* | *Mean* | *Standard*  *Deviation* | *Upper*  *95% ci* | *Lower*  *95% ci* |
| --- | --- | --- | --- | --- | --- | --- |
| T0 | Correct Information | 81 | 1.85 | 1.41 | 2.16 | 1.54 |
| T0 | Misleading Information | 81 | 1.83 | 1.51 | 2.16 | 1.49 |
| T1 | Correct Information | 81 | 1.67 | 1.47 | 2.00 | 1.35 |
| T1 | Misleading Information | 81 | 1.68 | 1.57 | 2.02 | 1.33 |
| T2 | Correct Information | 81 | 2.00 | 1.30 | 2.29 | 1.71 |
| T2 | Misleading Information | 81 | 1.94 | 1.23 | 2.21 | 1.66 |

The table shows the mean, the standard deviation, the 95% Confidence Intervals (Upper 95% CI, Lower 95% CI) of the score of Question 6 “it seemed like my hand was in the location where the virtual hand was”.

**
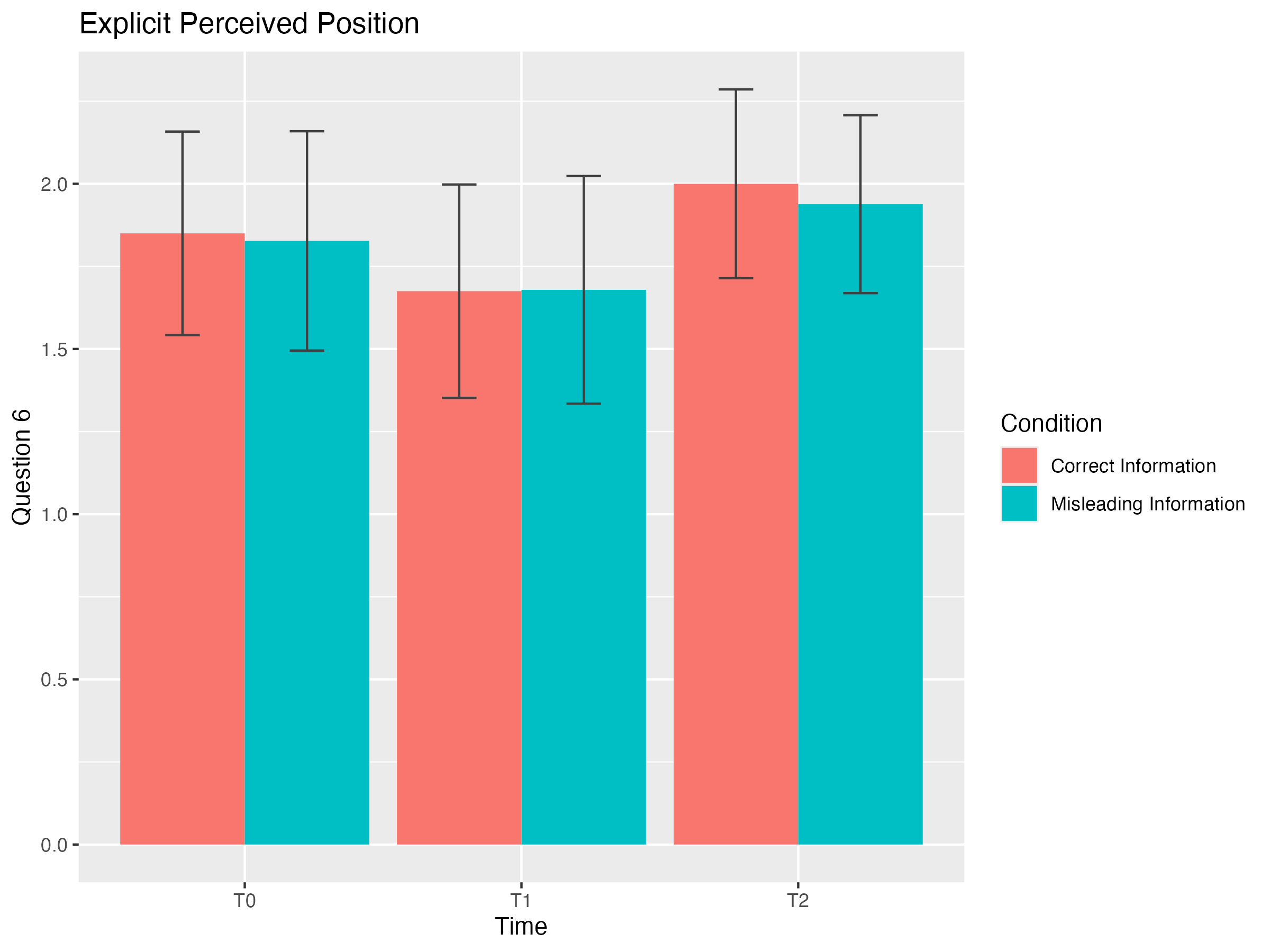
**

**Figure SI2**. The figure shows the mean values of Question 6. Mean scores are all positive and higher than 1.6, indicating that their hand position is perceived to be closer to the virtual hand. The error bars indicate 95 % Confidence intervals.

**Section SI1. Models and Posterior Predictive Check for each analysis performed.**

The file shows the model adopted for each analysis, specifying priors, family, chains and iterations.

**Bayesian Regression on Embodiment**

*Model*

brm_emb <- brm(

Embodiment ~ Time * Condition +

(1 + Time + Condition || ID),

data = total.db,

family = student,

prior = c( set_prior("normal(0,0.5)", class = "b"),

set_prior("normal(0,1)", class = "sd"),

set_prior("normal(0,0.5)", class = "Intercept", lb=-3, ub=3)),

sample_prior = T,

chains = 4,

iter = 8000,

cores = 15,

save_pars = save_pars(all=T)))

*Posterior predictive check*pp_check(brm_emb, ndraws = 500)

*
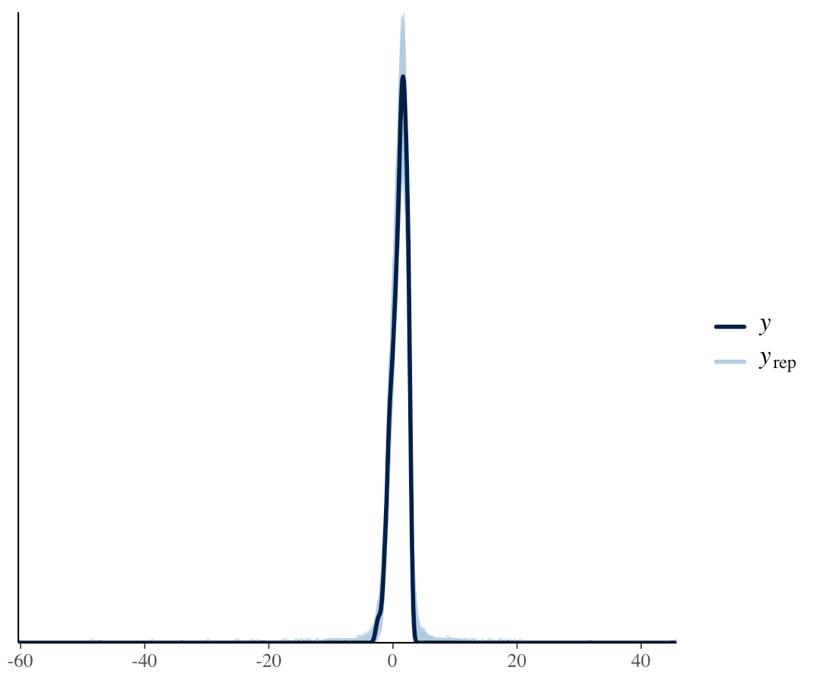
*

**Bayesian Regression on Disembodiment**

*Model*

brm_disemb <- brm(
 Disembodiment ~ Time * Condition+
 (1 + Time + Condition|| ID),
 data = total.db,
 family = skew_normal,
 prior = c( set_prior("normal(0,0.5)", class = "b"),

set_prior("normal(0,1)", class = "sd"),

set_prior("normal(0,0.5)", class = "Intercept", lb=-3, ub=3)),

sample_prior = T,
 chains = 4,
 iter = 8000,
 cores = 15,
 save_pars = save_pars(all=T))

*Posterior predictive check*
pp_check(brm_disemb, ndraws = 500)


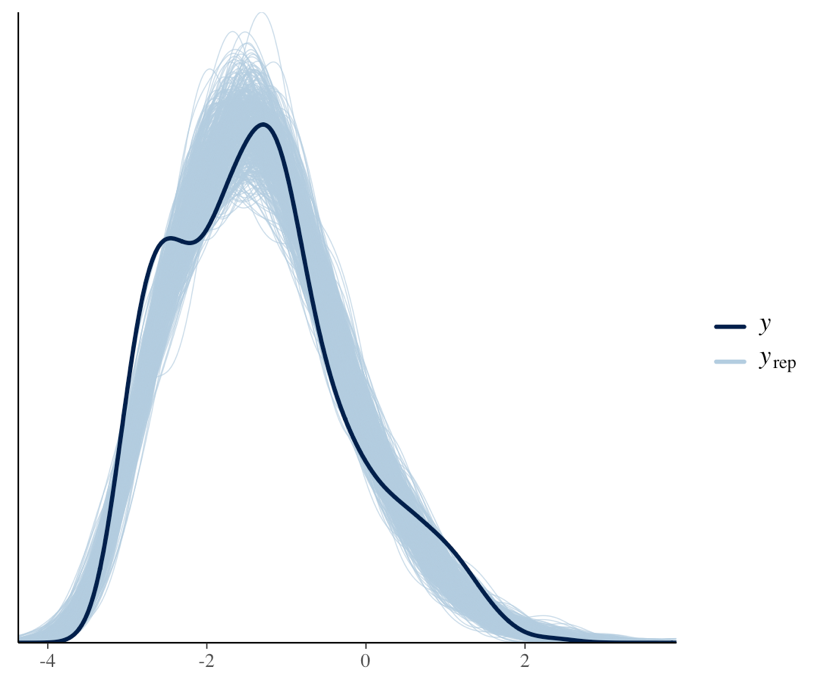


**Bayesian Regression on Physical Sensations**

The dependent variable distribution did not follow a Guassian shape but showed peaks around -3 and 0. Given that the cumulative distribution provided the most accurate fit for the data, we recode the variable. We transformed of the original scale from 1 to 7, thus creating an integer variable without zero. We used the default brm priors for this family distribution.

*Model*
brm_phys <- brm(
 PhysicR ~ Time * Condition+
 (1 + Time + Condition || ID),
 data = total.db,
 family = cumulative,

sample_prior = T,
 chains = 4,
 iter = 8000,
 cores = 15,
 save_pars = save_pars(all=T))

*Posterior predictive check*
pp_check(brm_phys, ndraws = 500)


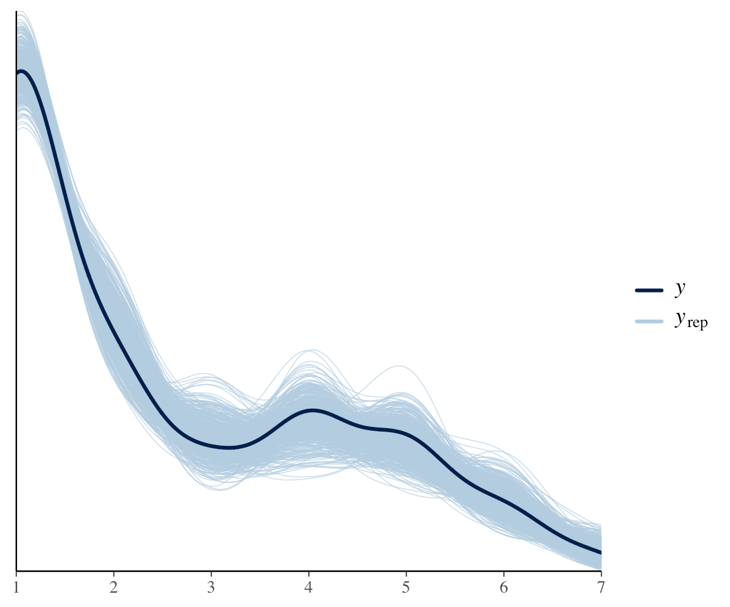


**Bayesian Regression on Proprioceptive Drift**

*Model*
brm_propD_trial <- brm(
 Prop.drift ~ Time * Condition +
 (1 + Time + Condition || ID + Trial),
 data = total.db.long,
 family = student,
 prior = c(set_prior("normal(0,0.5)", class = "b"),
 set_prior("normal(0,1)", class = "sd"),
 set_prior("normal(0,0.5)", class = "Intercept")),
 sample_prior = T,
 chains = 4,
 iter = 8000,
 cores = 15,
 save_pars = save_pars(all=T))

*Posterior predictive check*
pp_check(brm_propD_trial, ndraws = 500)


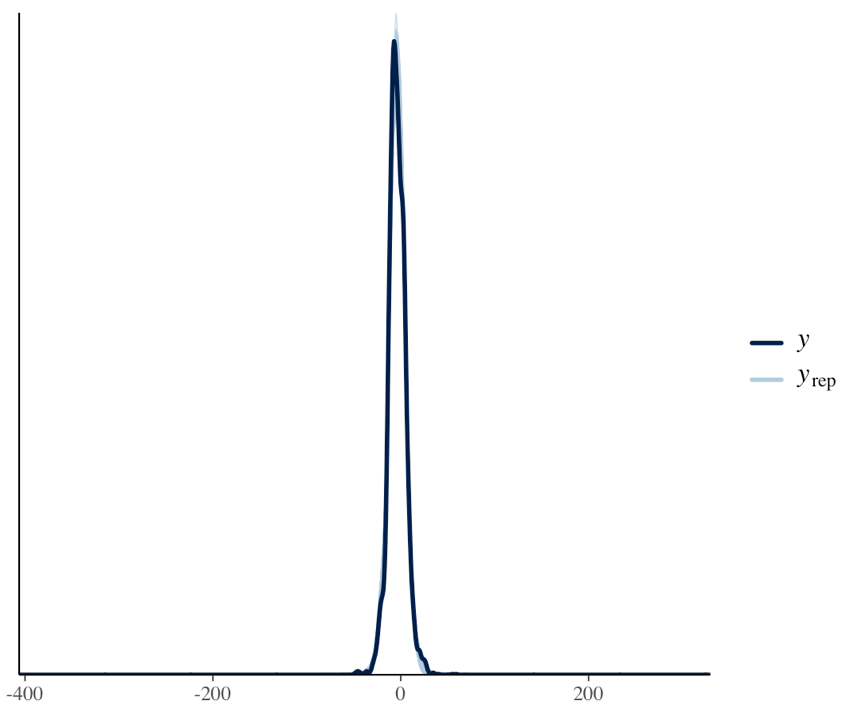


**Section SI2. Robustness Check**

**Table A.** **Bayesian regression on Embodiment with less (standard deviation = 1) and more (standard deviation = 0.3) informative priors.**

| **Embodiment Effects**  **SD = 1** | Estimate | Est. Error | Lower  95% CI | Upper  95% CI |
| --- | --- | --- | --- | --- |
| Intercept | **0.99** | **0.12** | **0.75** | **1.21** |
| Time_T1_ | -0.03 | 0.05 | -0.12 | 0.06 |
| Time_T2_ | **-0.30** | **0.05** | **-0.39** | **-0.22** |
| Condition_Misleading_ | 0.10 | 0.05 | -0.01 | 0.20 |
| Time_T1_*Condition_Misleading_ | 0.07 | 0.09 | -0.09 | 0.24 |
| Time _T2_* Condition_Misleading_ | -0.04 | 0.08 | -0.21 | 0.11 |
| BF for interaction inclusion Time×Condition | **0.009** | | | |
| **SD = 0.3** | *Estimate* | *Est. Error* | *Lower*  *95% CI* | *Upper*  *95% CI* |
| Intercept | **0.99** | **0.12** | **0.75** | **1.23** |
| Time_T1_ | -0.03 | 0.05 | -0.13 | 0.06 |
| Time_T2_ | **-0.30** | **0.05** | **-0.39** | **-0.21** |
| Condition_Misleading_ | 0.09 | 0.05 | -0.01 | 0.20 |
| Time_T1_*Condition_Misleading_ | 0.07 | 0.08 | -0.10 | 0.23 |
| Time _T2_* Condition_Misleading_ | -0.04 | 0.08 | -0.19 | 0.11 |
| BF for interaction inclusion Time×Condition | **0.061** | | | |

The table shows the mean (Estimate) and the standard deviation (Est. Error) of the posterior distribution of each effect with the 95% Credible Intervals (lower 95% CI, upper 95% CI) considering different priors. In bold, the posterior distributions without a zero overlapping. Bayes Factor (BF) for the inclusion of the interaction Time × Verbal Conditioning is also reported. Robustness checks confirmed the result across prior specifications: evidence consistently favored the model without the interaction, with BF_incl_ =0.009 with a less informative prior (standard deviation = 1) and BF_incl_ =0.061 with a more informative prior (standard deviation = 0.3).

**Table B.** **Bayesian regression on Disembodiment with less (standard deviation = 1) and more (standard deviation = 0.3) informative priors.**

**.**

| **Disembodiment Effects**  **SD = 1** | Estimate | | Est. Error | Lower  95% CI | Upper  95% CI |
| --- | --- | --- | --- | --- | --- |
| Intercept | **-1.27** | **0.10** | | **-1.47** | **-1.07** |
| Time_T1_ | 0.04 | 0.06 | | -0.08 | 0.17 |
| Time_T2_ | -0.03 | 0.05 | | -0.13 | 0.07 |
| Condition_Misleading_ | 0.01 | 0.06 | | -0.10 | 0.12 |
| Time_T1_*Condition_Misleading_ | -0.10 | 0.10 | | -0.31 | 0.10 |
| Time _T2_* Condition_Misleading_ | 0.09 | 0.10 | | -0.10 | 0.29 |
| BF for interaction inclusion Time×Condition | **0.001** | | | | |
| **SD = 0.3** | *Estimate* | *Est. Error* | | *Lower*  *95% CI* | *Upper*  *95% CI* |
| Intercept | **-1.28** | **0.11** | | **-1.49** | **-1.06** |
| Time_T1_ | 0.04 | 0.06 | | -0.08 | 0.16 |
| Time_T2_ | -0.03 | 0.05 | | -0.13 | 0.07 |
| Condition_Misleading_ | 0.01 | 0.05 | | -0.10 | 0.11 |
| Time_T1_*Condition_Misleading_ | -0.09 | 0.10 | | -0.28 | 0.10 |
| Time _T2_* Condition_Misleading_ | 0.08 | 0.09 | | -0.10 | 0.26 |
| BF for interaction inclusion Time×Condition | **0.011** | | | | |

The table shows the mean (Estimate) and the standard deviation (Est. Error) of the posterior distribution of each effect with the 95% Credible Intervals (lower 95% CI, upper 95% CI) considering different priors. In bold, the posterior distributions without a zero overlapping. Bayes Factor (BF) for the inclusion of the interaction Time × Verbal Conditioning is also reported. Robustness checks confirmed the result across prior specifications: evidence consistently favored the model without the interaction, with BF_incl_ = 0.001 with a less informative prior (standard deviation = 1) and BF_incl_ = 0.011 with a more informative prior (standard deviation = 0.3).

**Table C.** **Bayesian t-test on SCR-PP between with less (standard deviation = 1) and more (standard deviation = 0.3) informative priors.**

| Prior (r) | BF_01_ | Estimate | Est. Error | Lower  95% CI | Upper  95% CI |
| --- | --- | --- | --- | --- | --- |
| **SD = 0.3** | **2.39** | -0.09 | 0.11 | -0.31; | 0.11 |
| **SD = 1** | **6.66** | -0.11 | 0.11 | -0.34; | 0.12 |

The table shows Bayes Factor values to quantify the evidence for the absence of a difference between condition (BF_01_), the mean and the standard deviation of posterior distribution of the standardized paired difference with the 95% Credible Intervals (lower 95% CI, upper 95% CI) considering different priors. These results confirm moderate and robust evidence in favour of the null hypothesis, regardless of prior specification.

**Table D.** **Bayesian regression on Proprioceptive with less (standard deviation = 1) and more (standard deviation = 0.3) informative priors.**

| **Proprioceptive Drift Effects**  **SD = 1** | Estimate | Est. Error | Lower  95% CI | Upper  95% CI |
| --- | --- | --- | --- | --- |
| Intercept | **-1.34** | **0.42** | **-2.15** | **-0.50** |
| Time_T1_ | **1.26** | **0.35** | **0.58** | **1.94** |
| Time_T2_ | 0.16 | 0.31 | -0.45 | 0.75 |
| Condition_Misleading_ | **-1.09** | **0.40** | **-1.87** | **-0.29** |
| Time_T1_*Condition_Misleading_ | **1.02** | **0.24** | **0.55** | **1.49** |
| Time _T2_* Condition_Misleading_ | **-0.60** | **0.23** | **-1.05** | **-0.13** |
| BF for interaction inclusion Time*Condition | **777.487** | | | |
| **SD= 0.3** | *Estimate* | *Est. Error* | *Lower*  *95% CI* | *Upper*  *95% CI* |
| Intercept | **-1.34** | **0.43** | **-2.17** | **-0.48** |
| Time_T1_ | **0.55** | **0.24** | **0.08** | **1.03** |
| Time_T2_ | 0.10 | 0.22 | -0.33 | 0.53 |
| Condition_Misleading_ | **-0.40** | **0.25** | **-0.89** | **0.09** |
| Time_T1_*Condition_Misleading_ | **0.60** | **0.19** | **0.24** | **0.97** |
| Time _T2_* Condition_Misleading_ | **-0.29** | **0.18** | **-0.64** | **0.07** |
| BF for interaction inclusion Time*Condition | **74.538** | | | |

The table shows the mean (Estimate) and the standard deviation (Est. Error) of the posterior distribution of each effect with the 95% Credible Intervals (lower 95% CI, upper 95% CI) considering different priors. In bold, the posterior distributions without a zero overlapping. Bayes Factor (BF) for the inclusion of the interaction Time × Verbal Conditioning is also reported. Robustness checks confirmed our results across different priors, evidence strongly favored the model with the interaction, with BF_incl_ = 777.487 for a less informative prior (standard deviation = 1) and BF_incl_ = 74.538 for a more informative prior (standard deviation = 0.3)

**Table E. Effect of Virtual Drift in T1 on Virtual Drift in T2, regardless virtual drift in T0 with less (standard deviation = 1) and more (standard deviation = 0.3) informative priors.**

| Condition | Prior | BF | Estimate | Est. Err | Lower  95% CI | Upper  95% CI |
| --- | --- | --- | --- | --- | --- | --- |
| **Correct Information** | **SD = 1** | 1,4×10^4^ | 0.48 | 0.093 | 0.30 | 0.67 |
|  | **SD = 0.3** | 1,4 ×10^4^ | 0.48 | 0.095 | 0.30 | 0.67 |
| **Misleading Information** | **SD = 1** | 1,10×10⁸ | 0.71 | 0.103 | 0.51 | 0.91 |
|  | **SD = 0.3** | 9,13×10^6^ | 0.71 | 0.104 | 0.50 | 0.91 |

**Results of the Bayesian regression with virtual drift at T2 as dependent variable and virtual drift at T1 and T2 as predictors with different priors.** The table shows the Bayes Factor comparing the model against the intercept only model to quantify the strength of evidence for virtual drift in T1 as a predictor of embodiment in t2, net of virtual drift in T0. We extracted posterior estimates for the model parameters, reporting the mean (Estimate) and the standard deviation (Est. Error) of the posterior distribution with the 95% Credible Intervals (lower 95% CI, upper 95% CI) considering different priors. Robustness checks confirmed that the robust evidence supporting virtual drift at T1 as predictor of virtual drift at T2, net of virtual drift in t0, remained consistent across different prior specifications (standard deviation= 0.3, and standard deviation = 1).

**Table F. Effect of Virtual Drift in T1 on Embodiment Score in T2 between with less (standard deviation = 1) and more (standard deviation = 0.3) informative priors.**

| *Condition* | *Prior* | *BF_01_* | *Estimate* | *Est. Err.* | *Lower*  *95% CI* | *Upper*  *95% CI* |
| --- | --- | --- | --- | --- | --- | --- |
| *Correct Information* | **SD = 0.3** | 1.80 | -0.016 | 0.013 | -0.04 | 0.01 |
|  | **SD = 1** | 4.81 | -0.018 | 0.014 | -0.05 | -0.01 |
| *Misleading Information* | **SD = 0.3** | 3.74 | -0.001 | 0.015 | -0.03 | 0.03 |
|  | **SD = 1** | 11.33 | -0.001 | 0.017 | -0.03 | 0.03 |

The table shows Bayes Factor values is reported to quantify the evidence for the null model over the alternative (BF_01_), the mean (Estimate) and the standard deviation (Est. Error) of the posterior distribution with the 95% Credible Intervals (lower 95% CI, upper 95% CI) considering different priors. Robustness checks confirmed evidence supporting the absence of a relationship between virtual drift at T1 and embodiment at T2 remained consistent across different prior specifications. In the Correct Information condition, anecdotal evidence was found with a more informative prior (r = 0.3; BF₀₁ = 1.80), and moderate evidence emerged with a less informative prior (r = 1.0; BF₀₁ = 4.81). In the Misleading Information condition, results consistently indicated moderate evidence against T1 virtual drift as a predictor of T2 embodiment, both with a more informative prior (standard deviation = 0.3; BF₀₁ = 3.74) and a less informative prior (standard deviation = 1.0; BF₀₁ = 11.33).

**Section SI3. One-Sample t-tests on Embodiment and Proprioceptive Drift at T0**

Two specific hypotheses were tested at T0 using two Bayesian one-sample t-tests with a Cauchy prior (r = 0.707):

(i) Presence of embodiment sensations already after the visual exposure: H₁: mean embodiment > 0.

(ii) Rightward proprioceptive drift after the visual exposure: H₁: mean proprioceptive drift < 0

The Bayesian one-sample t-test on Embodiment scores at T0 provided evidence that Embodiment is higher than zero after the simple visual exposure to the virtual hand (BF₊ = 4.96 × 10¹⁵). The complementary direction (μ ≤ 0) received less support (BF₋= 3.96 × 10⁴). Comparing the two directional alternatives directly, the evidence favored the μ > 0 hypothesis (BF₊/BF₋ = 1.25 × 10¹¹). This result supports the idea that mere observation of a virtual hand, even when misaligned with the real body position, can already elicit embodiment sensations.

The Bayesian one-sample t-test on Proprioceptive Drift at T0 provided evidence that the shift is negative after the simple visual exposure to the virtual hand (BF₋ = 245.64). The complementary direction (μ ≥ 0) received no support (BF₊ = 0.0178). Directly comparing the two directional alternatives, the evidence favored the μ < 0 hypothesis (BF₊/BF₋ = 1.38 × 10⁴). Thus, mere visual exposure to the misaligned virtual hand already produced a rightward shift in perceived hand position (i.e., toward the virtual hand).
